# Clinical and molecular correlates of JAK-inhibitor therapy failure in myelofibrosis: long-term data from a molecularly annotated cohort

**DOI:** 10.1038/s41375-022-01544-x

**Published:** 2022-03-26

**Authors:** James T. England, Caroline J. McNamara, James A. Kennedy, Jose-Mario Capo-Chichi, Jingyue Huang, Andrea Arruda, Taylor Nye, Verna Cheung, Jaime O. Claudio, Dawn Maze, Hassan Sibai, Anne Tierens, Hubert Tsui, Aniket Bankar, Wei Xu, Tracy Stockley, Vikas Gupta

**Affiliations:** 1 Medical Oncology and Hematology, Princess Margaret Cancer Centre, Toronto, Ontario Canada; 2 Medical Oncology and Hematology, Sunnybrook Health Sciences Centre, Toronto, Ontario Canada; 3 Division of Clinical Laboratory Genetics, Laboratory Medicine Program, University Health Network, Toronto, Ontario Canada; 4 Biostatistics, Princess Margaret Cancer Centre, Toronto, Ontario Canada; 5 Laboratory Hematology, Toronto General Hospital, Toronto, Ontario Canada; 6 Department of Laboratory Medicine and Pathobiology, University of Toronto, Toronto, Ontario Canada

**Keywords:** Genetics research, Myeloproliferative disease

## To the Editor:

Myelofibrosis (MF) is an acquired clonal hematopoietic stem cell disorder associated with debilitating constitutional symptoms, extramedullary hematopoiesis resulting in splenomegaly, and a propensity to transform to a blast phase/acute myeloid leukemia (AML). The discovery of JAK inhibitors (JAKi) has been pivotal in the treatment of symptomatic MF by reducing spleen size, and alleviating cytokine-related symptom burden [1]. Despite this, up to 50% of MF patients discontinue JAKi by 2–3 years and only one quarter of patients remain on treatment at 5 years [2, 3].

Prospective trials of JAKi therapy provide little information after patients discontinue therapy and safety specific follow up is completed. Although survival following ruxolitinib cessation is poor, in the range 13–16 months, the clinical course and reasons for JAKi failure in MF patients are not well characterized [4–7]. Criteria for JAKi failure are variably defined in retrospective studies and second-line JAKi therapy trials [8–10]. JAKi therapy may fail for a variety of reasons including sub-optimal/loss of spleen response, severe cytopenias, progression to an accelerated or blast phase (AP/BP) of disease, secondary malignancies other than AML, recurrent severe infections, or other non-hematological toxicities. Recognition of patterns of failure is important to accurately characterize, and plan treatment strategies in these patients.

We conducted a retrospective study analyzing a molecularly annotated, mature dataset of MF patients treated with JAKi followed in a prospective MPN registry (NCT02760238) at Princess Margaret Cancer Centre. We evaluated the impact of baseline clinical and molecular factors on clinical outcomes and therapy failure. We characterized different patterns of JAKi failure according to consensus criteria of the Canadian MPN Group (Supp. Table S1) [11] and its impact on survival. In a sub-set of patients with paired samples we evaluated the impact of clonal evolution on outcomes following JAKi failure. Cohort selection, study definitions, molecular, and statistical analysis are summarized in Appendix.

After search of our MPN database and exclusion of ineligible patients (Supp. Fig. S1), 113 patients with a diagnosis of MF in chronic phase treated with JAKi along with a sample for mutation analysis were included. The baseline patient, disease, and treatment related characteristics of the study population are summarized in Supp. Table S2.

During the course of the study period 85 (75%) patients died with median follow-up in survivors of 74 (range: 21–120) months. A total of 107 (95%) patients experienced JAKi failure; and cumulative incidence of JAKi failure at 1, 3, and 5 years was 34%, 71%, 87%, respectively (Supp. Fig. S2). Multivariable analysis is summarized in Supp. Table S3 for both cumulative JAKi failure and OS from JAKi initiation. ECOG performance status and *CBL* mutation demonstrated significant predicative value for both JAKi failure and OS from JAKi initiation; while the number of mutations predicted OS but had no significant effect on probability of JAKi failure. Platelet count did not predict either JAKi failure or OS, while transfusion requiring anemia (RBC Tx) was predictive for OS in model 1, and JAKi failure in both models.

**Fig. 1:**
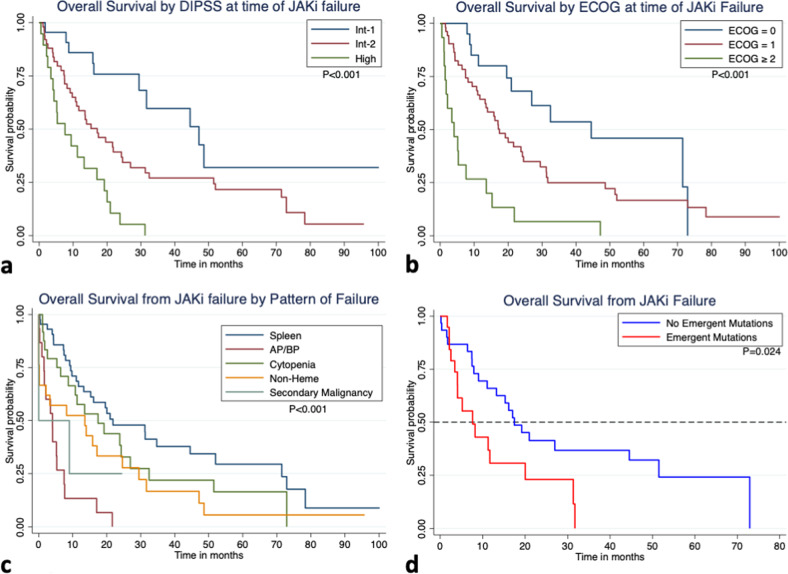
Predictors of overall survival following JAKi failure. Kaplan-Meier survival curve for **a** DIPSS at the time of JAKi failure, **b** ECOG at time of failure, **c** clinical pattern of JAKi failure, and **d** presence of emergent mutations. Survival curves compared with log rank method.

The clinical features at time of JAKi failure are summarized in Supp. Table S4. In MVA (Supp. Table S5) failure from AP/BP disease, high DIPSS, and ECOG ≥ 2 were significantly associated with inferior survival following JAKi failure (Fig. 1a–c).

**Fig. 2:**
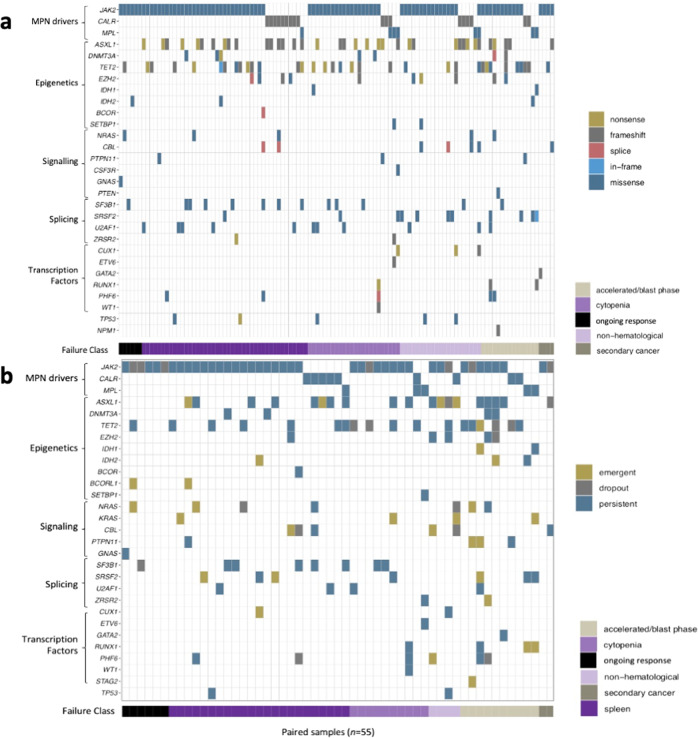
Geneplots for patients treated with JAKi organized by pattern of JAKi failure (x-axis) and mutation category (y-axis). **a** Baseline mutations present prior to start of JAKi therapy, with no difference in number or genes mutated between pattern of failure groups. **b** Geneplot demonstrating *n* = 55 patients with paired mutation analysis arranged by pattern of failure (*n* = 49) or ongoing response to JAKi (*n* = 6). Emergent mutations were more frequently observed in patients with JAKi failure due to AP/BP (*n* = 7/10) than failure due to cytopenia (*n* = 1/10, *P* = 0.006).

The patterns of JAKi failure were as follows: sub-optimal (*n* = 8, 7%) or loss (*n* = 35, 33%) of spleen response (*n* = 43 total, 40%); cytopenias (*n* = 24, 22% total; thrombocytopenia = 14, 13%; transfusion dependence = 10, 9%); AP/BP transformation (*n* = 15, 14%); non-hematological toxicity (*n* = 21, 20%), and second malignancy (*n* = 4, 4%). For all patients (*n* = 107), median [95%CI] OS following JAKi failure was 13.6 [9.0–19.6] months. Median survival by pattern of failure was: 4.1 [1.0–5.3] months for AP/BP; 17.5 [6.5–27.0] months for cytopenias; 21.8 [11.7–44.5] months for loss of/suboptimal spleen response; 0.3 [0.3- not reached] months for second malignancy and 13.6 [0.2–24.6] months for non-hematological toxicity (*p* < 0.001). There was no association between baseline mutations, number of mutations, or number of HMR mutations and pattern of JAKi failure (Fig. 2a).

Analyis of paired sequencing was performed on 55 patients (Fig. 2b) who had a later molecular sample available, either at the time of JAKi failure (*n* = 49) or after at least 3-years sustained clinical response to JAKi therapy (*n* = 6). Of the six patients with ongoing benefit from therapy: three patients had the same variants detected, two patients had dropout of mutations, and one patient had two emergent mutations (*NRAS, BCORL1*) and dropout of *JAK2*.

At time of JAKi failure 24 (49%) patients had no change in observed variants. Dropout of 18 previously identified variants in 12 (24%) patients was observed; with dropout of *JAK2* (*n* = 4), and *TET2* (*n* = 4) the most commonly observed. A total of 29 emergent mutations were observed in 19 (39%) patients. The most common emergent mutations were in *KRAS* (*n* = 4) and *ASXL1* (*n* = 4); with RAS pathway genes (*KRAS, NRAS, CBL*, and *PTPN11*) and HMR the most common class of emergent mutations occurring in 9 (47%) patients each. Emergent mutations were more frequently observed in patients with JAKi failure due to AP/BP (*n* = 7/10, 70%) than failure due to cytopenia (n = 1/10, 10%, *P* = 0.006); while there was no significant difference when compared to patients with failure due to loss or lack of spleen response (*n* = 8/23, 35%), non-hematological toxicity (*n* = 3/4, 75%), or secondary malignancy (*n* = 0/2, 0%). The median overall survival following JAKi failure was significantly shorter in those with emergent mutations compared to those without (*p* = 0.02, Fig. 1d).

This study provides further understanding of the clinical and molecular outcomes following JAKi failure. Our analysis differs from previous studies looking at outcomes after JAKi discontinuation, as we used standardized JAKi failure definitions as opposed to relying on drug discontinuation as the sole indicator of failure. Despite this key difference, the overall survival following JAKi failure is poor and similar to previous reports [4–7]. In MVA clinical variables including ECOG performance status, RBC Tx, and molecular factors including *CBL* and total number of mutations predict OS independent of MIPSS risk category. A shorter time to JAKi failure was predicted by ECOG, RBX Tx, and *CBL* mutation; though not by MIPSS or total number of mutations. These results add further evidence and validation for the consideration of *CBL* mutations in future revision of the definition of high-risk MF [12].

Our study has expanded on previous research by describing and analyzing clinical features, correlates, and outcomes according to the pattern of JAKi failure. Patients who develop AP/BP or non-hematological malignancy on JAKi have a dismal prognosis; while outcomes following spleen progression or cytopenias have comparable outcomes. It is also important to note that in clinical practice, these failure reasons do not occur in isolation. For example, a patient may develop cytopenias requiring a dose reduction in JAKi and as a result the patient then loses their spleen and/or symptom response. Adherence to standardized criteria of JAKi failure will help in early recognition of the pattern of failure, facilitate clinical trial enrollment, and understanding of comparative effectiveness of novel agents.

The emergence of mutations in our cohort was common, occurring in 37% of patients. This contrasts with previous reports from Lundberg et al., which detected only two new mutations in chronic MPN patients during 133 patient-years follow-up [13]. The difference in observed mutation rate may be in part due to patient population, as that study had <20% of the cohort comprised of MF patients. The population of MF patients requiring JAKi therapy may have more advanced disease and molecular complexity compared to those not requiring pharmacologic intervention.

The clinical significance of clonal evolution as evidenced by the emergence of new mutations on paired analysis is an area of ongoing research. Consistent with previous reports, our data demonstrate that variant emergence is associated with inferior survival following JAKi failure [5, 14]. Baseline mutation profile did not predict the pattern of JAKi failure; though emergent mutations were noted to be more common amongst patients with AP/BP. Our study had frequent emergent *ASXL1* mutations (21% of patients with emergent mutations) similar to a study from MD Anderson [5], but there were also frequent emergent mutations in the RAS pathway (47% of patients with emergent mutations overall; 21% *KRAS*). Differences between these cohort studies may be due to use of JAKi failure rather than drug discontinuation as our endpoint; or the larger panel of genes evaluated by NGS used in our study in particular with the inclusion of *CBL* [5, 15]. Our data suggest that detection of newly emergent mutations at the time of JAKi failure may further inform poor prognosis, with mutations in RAS pathway and HMR genes frequently observed at time of failure. How activating mutations in alternative growth signaling pathways such as RAS may influence resistance to JAKi and subsequent outcomes with second-line therapies warrants further investigation.

In conclusion, we demonstrate that outcomes following JAKi failure are significantly correlated with the pattern of failure. Patients who transform to AP/BP have dismal outcomes, where as those with sub-optimal or loss of response or significant cytopenias have similar outcomes. Baseline molecular signatures did not predict the pattern of JAKi failure; however, development of emergent mutation at time of JAKi failure is observed more frequently with AP/BP disease.

## Supplementary information

Supplementary material
