# Supplementary material for: Clinical and molecular correlates of JAK-inhibitor therapy failure in myelofibrosis: long-term data from a molecularly annotated cohort
Source: Leukemia. 2022 Mar 26;36(6):1689–92. doi: 10.1038/s41375-022-01544-x (PMC9162913; doi:10.1038/s41375-022-01544-x)
Supplement: Supplementary file 1 — Supplementary material [file 41375_2022_1544_MOESM1_ESM.pdf]

**Appendix**

*Population*

All patients provided written informed consent for banking of a molecular sample. Ethics approval was obtained from the University Health Network Research Ethics Board. Eligibility criteria required: (i) diagnosis of chronic phase (<10% blasts in peripheral blood or bone marrow) primary MF, post-polycythemia vera MF (PPV MF) or post-essential thrombocythemia MF (PET MF), (ii) received first line JAKi therapy with either ruxolitinib or momelotinib, and (iii) have a molecular sample available for analysis at the time of starting JAKi. For paired sequencing analysis, patients were included if they had available molecular sample at the time of: (i) JAKi failure or (ii) after ≥3 years in patients who continue to benefit from JAKi therapy.

*Definitions*

Mutations *in ASXL1, EZH2, IDH1/2, SRSF2,* or *U2AF1 Q157* were classified as high molecular risk (HMR) based on prior reports(1, 2). The Mutation-Enhanced International Prognostic Score System (MIPSS70, http://www.mipss70score.it/) risk categories were calculated for patient at time of JAKi initiation(1, 3). Dynamic International Prognostic Scoring System (DIPSS) scores were calculated on the first day of JAKi and on clinical assessment following JAKi failure(4). Cytogenetic results were assessed from the most recent peripheral blood or bone marrow sample prior to starting JAKi.

Criteria for JAKi failure was documented according to the Canadian consensus criteria as listed in Supp. Table S1(5).

*Molecular Analysis*

Targeted Next-Generation Sequencing (NGS, paired end sequencing, Miseq v2, Illumina, San Diego, CA) was performed on high-quality DNA extracted from peripheral blood or bone

marrow samples. NGS was carried out using one of two NGS panels 1) the Trusight Myeloid amplicon-based panel (Illumina, 54 myeloid genes, Supp. Table S6) from samples collected until April 2018, or 2) a custom hybridization capture-based panel (Oxford Gene Technologies, 49 myeloid genes, Supp. Table S7) for samples collected from April 2018 onward. Sequencing reads were processed as previously described(6-8). Landscape plots were prepared using the GenVisR R package (Skidmore et al, Bioinformatics 2016).

For the comparisons between paired samples, genes not included on both panels were excluded and variants were only considered significant if present with an allele frequency (VAF) >5% to account for methodologic and sensitivity differences between the two panels. Pathogenic/likely pathogenic variants were classified as persistent (present at >5% VAF at both time points), emergent (only present at >5% VAF at the later time point) or dropout (>5% VAF at the first time point only).

*Statistical analysis*

Descriptive statistics were provided with median and range for continuous variables, and frequencies and percentages for categorical variables. To compare different subgroups, Kruskal-Wallis test was used for continuous variables, and chi-square test and Fisher exact test were used for categorical variables. Time to JAKi failure was defined as time from start of JAKi therapy to treatment failure meeting the criteria of the Canadian MPN Group or date of last follow-up. Overall Survival (OS) time was defined as time from either initiation of or failure of JAKi therapy to date of death or last follow-up. Univariate and multivariate Cox proportional hazards regression models were used to assess the association of clinical, and molecular factors on cumulative incidence of JAKi failure and OS. Mutations in genes that were present in ≥5% of the patient population were included in univariate analysis. Multivariable analysis (MVA) was

conducted using clinical and molecular variables that had P<0.10 on univariate analysis. Models for time to treatment failure and OS from JAKi initiation were based on the inclusion of MIPSS70; and variables included within those scores were not included separately within the models. The first MVA model (Model 1) included mutations as separate, independent variables; while Model 2 evaluated the impact of the total number of mutations. For analysis of OS after JAKi failure the MVA model was based on the DIPSS at time of failure.  Hazard ratio (HR) and corresponding 95% confidence interval (CI) were provided. Survival and JAKi failure probabilities were calculated using Kaplan-Meier (K-M) method. Differences in K-M survival and cumulative incidence curves were evaluated by log-rank test and results were considered significant if the p-value was less than or equal to 0.05. Statistical analyses were performed using SAS 9.4, Stata v.17, and R (R Foundation, Vienna, Austria).

**Supp. References**

1. Tefferi A, Guglielmelli P, Lasho TL, Gangat N, Ketterling RP, Pardanani A, et al. MIPSS70+ version 2.0: mutation and karyotype-enhanced international prognostic scoring system for primary myelofibrosis. Journal of clinical oncology: official journal of the American Society of Clinical Oncology. 2018;36(17):1769-70.
2. Vannucchi A, Lasho T, Guglielmelli P, Biamonte F, Pardanani A, Pereira A, et al. Mutations and prognosis in primary myelofibrosis. Leukemia. 2013;27(9):1861-9.
3. Guglielmelli P, Lasho TL, Rotunno G, Mudireddy M, Mannarelli C, Nicolosi M, et al. MIPSS70: mutation-enhanced international prognostic score system for transplantation-age patients with primary myelofibrosis. Journal of Clinical Oncology. 2018;36(4):310-8.
4. Passamonti F, Cervantes F, Vannucchi AM, Morra E, Rumi E, Pereira A, et al. A dynamic prognostic model to predict survival in primary myelofibrosis: a study by the IWG-MRT (International Working Group for Myeloproliferative Neoplasms Research and Treatment). Blood. 2010;115(9):1703-8.
5. Gupta V, Cerquozzi S, Foltz L, Hillis C, Devlin R, Elsawy M, et al. Patterns of Ruxolitinib Therapy Failure and Its Management in Myelofibrosis: Perspectives of the Canadian Myeloproliferative Neoplasm Group. JCO Oncology Practice. 2020:JOP. 19.00506.
6. Gupta V, Kennedy JA, Capo-Chichi J-M, Kim S, Hu Z-H, Alyea EP, et al. Genetic factors rather than blast reduction determine outcomes of allogeneic HCT in BCR-ABL–negative MPN in blast phase. Blood advances. 2020;4(21):5562-73.
7. Spiegel JY, McNamara C, Kennedy JA, Panzarella T, Arruda A, Stockley T, et al. Impact of genomic alterations on outcomes in myelofibrosis patients undergoing JAK1/2 inhibitor therapy. Blood advances. 2017;1(20):1729-38.
8. McNamara CJ, Panzarella T, Kennedy JA, Arruda A, Claudio JO, Daher-Reyes G, et al. The mutational landscape of accelerated-and blast-phase myeloproliferative neoplasms impacts patient outcomes. Blood advances. 2018;2(20):2658-71.

**Supp. Table S1:** Canadian MPN group's operational definition of ruxolitinib failure

| **Pattern of JAKi therapy failure** | **Definition** |
|---|---|
| Sub-optimal spleen response | <25% reduction in palpable spleen length after at least 3 months of optimally dosed JAKi therapy |
| Loss of spleen response | ≥50% increase in spleen length from best response |
| Transfusion dependent anemia | ≥4 units of red cell transfusions in 8 weeks occurring ≥ 6 months from ruxolitinib treatment |
| Severe thrombocytopenia | Unable to maintain unsupported platelet count >50 x $10^9$ /L in patients on anticoagulation; and >25 x $10^9$ /L in patients without anticoagulation |
| Transformation to AP/BP | Peripheral blood or bone blasts of ≥10% or biopsy proven myeloid sarcoma |
| Second Cancers | Diagnosis of a cancer (excluding MPN-related AML) after initiation of JAKi |

Abbreviations: JAKi, JAK inhibitor; Hb, hemoglobin; AML, acute myeloid leukemia

| **Supp. Table S2:** Baseline clinical, demographic and molecular data for myelofibrosis patients at the time of starting JAK Inhibitor therapy | |
|---|---|
| | **All Patients (n=113)** |
| **Age,** Median (range), years | 68 (42-86) |
| **Male,** n (%) | 70 (62) |
| **Hb,** Median (range), g/L | 97 (64-158) |
| **WBC,** Median (range), $x10^9/L$ | 14.4 (1.6-89.0) |
| **Plt count,** Median (range), $x10^9/L$ | 196 (17-1345) |
| **PBB** Median (range) | 1 .0(0.0-8.0) |
| **PBB** ≥1%, n (%) | 62 (55) |
| **RBC transfusion-requiring anemia (RBC Tx)**, n (%) | 45 (40) |
| **Spleen size (palpable),** Median (range), cm | 16.0 (3.5-38.0) |
| **ECOG,** n (%) | |
| 0 | 24 (22) |
| 1 | 77 (69) |
| ≥2 | 10 (9) |
| Missing | 2 |
| **Cytogenetics**, n (%) | |
| Normal | 52 (46) |
| Abnormal, favorable* | 24 (21) |
| Abnormal, not favorable** | 6 (5) |
| Failed, not done | 31 (27) |
| **DIPSS**, n (%) | |
| Low/ Intermediate-1[†] | 29 (26) |
| Intermediate-2 | 50 (44) |
| High | 34 (30) |
| **MIPSS70**, n (%) | |
| Low/Intermediate[‡] | 40 (35) |
| High | 73 (65) |
| **JAK Inhibitor,** n (%) | |
| Ruxolitinib | 85 (75) |
| Momelotinib | 28 (25) |
| **Driver Mutations**[¥], n (%) | |
| *JAK2* | 88 (78) |
| *MPL* | 8 (7) |
| *CALR* | 19 (17) |
| **Non-MPN Driver mutations (≥5 patients)** | |
| ***ASXL1*,** n (%) | 37 (33) |
| ***TET2*,** n (%) | 33 (29) |
| ***SF3B1*,** n (%) | 13 (12) |
| ***SRSF2*,** n (%) | 12 (11) |
| ***U2AF1*,** n (%) | 11 (10) |
| ***EZH2*,** n (%) | 10 (9) |

| | |
|---|---|
| ***CBL*****,** n (%) | 7 (6) |
| ***DNMT3A***, n (%) | 7 (6) |
| ***IDH1/ IDH2*****,** n (%) | 5 (4) |
| ***TP53*****,** n (%) | 5 (4) |
| **HMR mutations§,** n (%) | |
| No | 61 (54) |
| Yes | 52 (46) |
| **Number of mutations including Driving mutations,** n (%) | |
| 0-1 | 22 (19) |
| 2 | 48 (43) |
| ≥ 3 | 43 (38) |

*Sole abnormalities of 20q-, 13q-, +9, chromosome 1 translocation/duplication, -Y or sex chromosome abnormality other than –Y

**All other abnormalities

†Includes 1 patient with DIPSS “low” risk category

‡Includes 2 patient with MIPSS70 “low” risk category

¥Includes 1 patient with both *CALR* and *MPL* driver mutations

§Mutation in any of: *ASXL1*, *EZH2*, *IDH1/2*, *SRSF2*, or *U2AF1* Q157

| **Supp. Table S3:** Multivariable models for variables associated with JAKi failure and OS | | | | | | | |
|---|---|---|---|---|---|---|---|
| | **N** | **Cumulative Incidence of JAKi Failure** | | | **Overall Survival** | | |
| | | **HR(95%CI)** | **p-value** | **Global p-value** | **HR(95%CI)** | **p-value** | **Global p-value** |
| **Model 1** | | | | | | | |
| **MIPSS70** | | | | 0.06 | | | **0.004** |
| Low/ Intermediate | 40 | reference | | | reference | | |
| High | 73 | 1.5 (0.98,2.28) | | | 2.05 (1.26,3.33) | | |
| **Age > median** | 56 | 0.79 (0.52-1.22) | | 0.29 | 1.07 (0.66,1.72) | | 0.79 |
| **RBC Tx** | 45 | 2.31 (1.45,3.69) | | **<0.001** | 1.69 (1.06,2.7) | **0.03** | |
| **ECOG** | | | | **0.01** | | | **0.003** |
| 0 | 24 | reference | | | reference | | |
| 1 | 77 | 0.78 (0.46,1.32) | 0.36 | | 1.29 (0.69,2.43) | 0.42 | |
| ≥2 | 10 | 2.37 (1.04,4.94) | **0.03** | | 4.14 (1.71,10.05) | **0.002** | |
| *CBL* mutation | 7 | 3.13 (1.38,7.09) | **0.006** | | 4.31 (1.7,10.91) | **0.002** | |
| **Model 2** | | | | | | | |
| **MIPSS70** | | | | 0.15 | | | **0.03** |
| Low/ Intermediate | 40 | reference | | | reference | | |
| High | 73 | 1.37 (0.9,2.1) | | | 1.72 (1.05,2.81) | | |
| **Age > median** | 56 | 0.79 (0.51,1.23) | | 0.3 | 1.25 (0.78,2) | | 0.36 |
| **RBC Tx** | 45 | 2.06 (1.3,3.28) | | **0.002** | 1.41 (0.89,2.23) | 0.15 | |
| **ECOG** | | | | **0.01** | | | **0.03** |
| 0 | 24 | reference | | | reference | | |
| 1 | 77 | 0.75 (0.44,1.27) | 0.29 | | 1.31 (0.71,2.44) | 0.39 | |
| ≥2 | 10 | 2.18 (0.97,4.87) | 0.06 | | 3.08 (1.3,7.33) | **0.01** | |
| **Number Mutations** | | | | 0.24 | | | **0.007** |
| 0-1 | 22 | reference | | | reference | | |
| 2 | 48 | 1.16 (0.64,2.13) | 0.62 | | 1.84 (0.88,3.87) | 0.11 | |
| ≥3 | 43 | 1.59 (0.85,2.97) | 0.14 | | 3.00 (1.44,6.24) | **0.003** | |

| **Supp. Table S4:** Clinical features at time of JAKi Failure (n=107) | |
|---|---|
| **DIPSS** | n (%) |
| Not available* | 16 |
| Available | 91 |
| Intermediate-1 | 22 (24) |
| Intermediate-2 | 50 (55) |
| High | 19 (21) |
| **ECOG** | |
| Not available** | 19 |
| Available | 86 |
| 0 | 21 (24) |
| 1 | 52 (60) |
| ≥2 | 15 (17) |
| **Pattern of Failure** | |
| Spleen | 43 (40) |
| Suboptimal response† | 8 (7) |
| Loss of response‡ | 35 (33) |
| Significant Cytopenia | 24 (22) |
| Transfusion requiring anemia¥ | 10 (9) |
| Thrombocytopenia§ | 14 (13) |
| Acceleration/Blast phase | 15 (14) |
| Non-hematologic toxicity˜ | 21 (20) |
| Secondary malignancy | 4 (4) |

*****Death within 4 weeks of JAKi failure n=12, not captured n=4

******Death within 4 weeks of JAKi failure n=12, not captured n=7

†<25% reduction in palpable spleen length after at least 3 months of optimally dosed JAKi therapy

‡ ≥50% increase in spleen length from best response

¥≥4 units of red cell transfusions in 8 weeks occurring ≥ 6 months from JAKi treatment

§Unable to maintain unsupported platelet count >50 x $10^9$ /L in patients on anticoagulation; and >25 x $10^9$ /L in patients without anticoagulation

˜infection (n=6), neuropathy (n=3), renal impairment/injury (n=3), fatigue (n=2), headache (n=2), Liver injury (n=1), allergy (n=1), hemorrhage (n=1), peritonitis (n=1), arthralgia (n=1).

| **Supp. Table S5:** Multivariable analysis of clinical variables associated with survival following JAKi failure (n=107) | | | | |
|---|---|---|---|---|
| **Covariate** | **Number (%)** | **HR(95%CI)** | **p-value** | **Global p-value** |
| **DIPSS at failure** | 91 | | | **0.011** |
| Low/Int-1 | 22 (24) | reference | | |
| Int-2 | 50 (55) | 2 (0.95,4.22) | 0.07 | |
| High | 19 (21) | 4.06 (1.61,10.21) | **0.003** | |
| **ECOG at failure** | 86 | | | <**0.001** |
| 0 | 21 (24) | reference | | |
| 1 | 52 (60) | 1.65 (0.82,3.3) | 0.16 | |
| ≥2 | 15 (17) | 5.58 (2.27,13.72) | <**0.001** | |
| **Pattern of Failure** | 107 | | | 0.17 |
| Spleen | 43 (40) | reference | | |
| Cytopenia | 15 (14) | 1.52 (0.83,2.8) | 0.18 | |
| Acceleration/Blast Phase | 24 (22) | 2.44 (1.04,5.7) | **0.04** | |
| Other | 25 (23) | 1.11 (0.51,2.4) | 0.8 | |
| Non-hematologic | 21 (20) | | | |
| Secondary malignancy | 4 (4) | | | |

**Supp. Table S6: TruSight Myeloid (Illumina) NGS Gene Panel.** Genes and exon coverage in the TruSight Myeloid (Illumina) NGS Gene Panel

| Complete coding region coverage (15/54) | Hotspot coverage (39/54) | Exon Coverage |
|---|---|---|
| *BCOR* | *ABL1* | 4-6 |
| *BCORL1* | *ASXL1* | 12 |
| *CDKN2A* | *ATRX* | 8-10 and 17-31 |
| *CEBPA* | *BRAF* | 15 |
| *CUX1* | *CALR* | 9 |
| *DNMT3A* | *CBL* | 8, 9 |
| *ETV6/TEL* | *CBLB* | 9, 10 |
| *EZH2* | *CBLC* | 9, 10 |
| *IKZF1* | *CSF3R* | 14-17 |
| *KDM6A* | *FBXW7* | 9-11 |
| *PHF6* | *FLT3* | 14, 15, 20 |
| *RAD21* | *GATA1* | 2 |
| *RUNX1* | *GATA2* | 2-6 |
| *STAG2* | *GNAS* | 8, 9 |
| *ZRSR2* | *HRAS* | 2, 3 |
| | *IDH1* | 4 |
| | *IDH2* | 4 |
| | *JAK2* | 12, 14 |
| | *JAK3* | 13 |
| | *KIT* | 2, 8-11, 13, 17 |
| | *KMT2A* | 2, 3 |
| | *KRAS* | 5–8 |
| | *MPL* | 10 |
| | *MYD88* | 3-5 |
| | *NOTCH1* | 26-28, 34 |
| | *NPM1* | 12 |
| | *NRAS* | 2, 3 |
| | *PDGFRA* | 12, 14, 18 |
| | *PTEN* | 5, 7 |
| | *PTPN11* | 3, 13 |
| | *SETBP1* | 4 (partial) |
| | *SF3B1* | 13-16 |
| | *SMC1A* | 2, 11, 16, 17 |
| | *SMC3* | 10, 13, 19, 23, 25, 28 |
| | *SRSF2* | 1 |
| | *TET2* | 3-11 |
| | *TP53* | 2-11 |
| | *U2AF1* | 2, 6 |
| | *WT1* | 7, 9 |

**Supp. Table S7:** Genes covered by a custom hybridization capture-based panel (Oxford Gene Technologies, 49 myeloid genes)

| Complete coding sequence coverage (*n*=21) | | Hotspot gene region coverage (*n*=28) | | |
|---|---|---|---|---|
| **Gene** | **Transcript** | **Gene** | **Transcript** | **Target Exon** |
| BCOR | NM_001123385.1 | ASXL1 | NM_015338.5 | 13 |
| BCORL1 | NM_021946.4 | BRAF | NM_004333.4 | 15 |
| CEBPA | NM_004364.3 | CALR | NM_004343.3 | 9 |
| CTNNA1 | NM_001903.3 | CBL | NM_005188.3 | 8,9 |
| CUX1 | NM_001202543.1 | CSF3R | NM_156039.3 | 14-17 |
| DDX41 | NM_016222.3 | FBXW7 | NM_033632.3 | 9-11 |
| DNMT3A | NM_022552.4 | FLT3 | NM_004119.2 | 14,15,20 |
| ETNK1 | NM_018638.4 | GATA2 | NM_032638.4 | 2-6 |
| ETV6/TEL | NM_001987.4 | GNAS | NM_000516.4 | 8,9 |
| EZH2 | NM_004456.4 | IDH1 | NM_005896.3 | 4 |
| IKZF1 | NM_006060.5 | IDH2 | NM_002168.3 | 4 |
| IRF1 | NM_002198.2 | JAK2 | NM_004972.3 | 12,14 |
| JAK1 | NM_001321853.1 | KIT | NM_000222.2 | 2, 8-11,13,17 |
| PAX5 | NM_016734.2 | KMT2A/MLL | NM_001197104.1 | 5-8 |
| PHF6 | NM_032458.2 | KRAS | NM_033360.3 | 2,3 |
| PPM1D | NM_003620.3 | MPL | NM_005373.2 | 10 |
| RAD21 | NM_006265.2 | MYD88 | NM_002468.4 | 3,5 |
| RUNX1 | NM_001754.4 | NOTCH1 | NM_017617.3 | 26-28,34 |
| SH2B3/LNK | NM_005475.2 | NPM1 | NM_002520.6 | 12 |
| STAG2 | NM_001042749.2 | NRAS | NM_002524.3 | 2,3 |
| ZRSR2 | NM_005089.3 | PTPN11 | NM_002834.3 | 3,13 |
| | | SETBP1 | NM_015559.2 | 4 |
| | | SF3B1 | NM_012433.2 | 13-16 |
| | | SRSF2 | NM_001195427.1 | 1 |
| | | TET2 | NM_001127208.2 | 3-11 |
| | | TP53 | NM_000546.5 | 2-11 |
| | | U2AF1 | NM_001025203.1 | 2,6 |
| | | WT1 | NM_024426.3 | 7,9 |

**Figure Legends:**

**Supp. Figure S1:** Flow diagram showing selection of study cohort

**Supp. Figure S2:** Cumulative incidence of JAKi failure (a) whole cohort and stratified by (b) transfusion requiring anemia, (c) ECOG performance status, and d) mutation status of CBL. Failure incidence curves compared with log rank test.

**Supp. Figure S3:** Overall survival by (a) MIPSS70 risk category, (b) transfusion requiring anemia (c) CBL mutation status, and (d) number of mutations identified. Survival curves compared with log rank test.

**Supp. Figure S1:** Flow diagram showing selection of study cohort

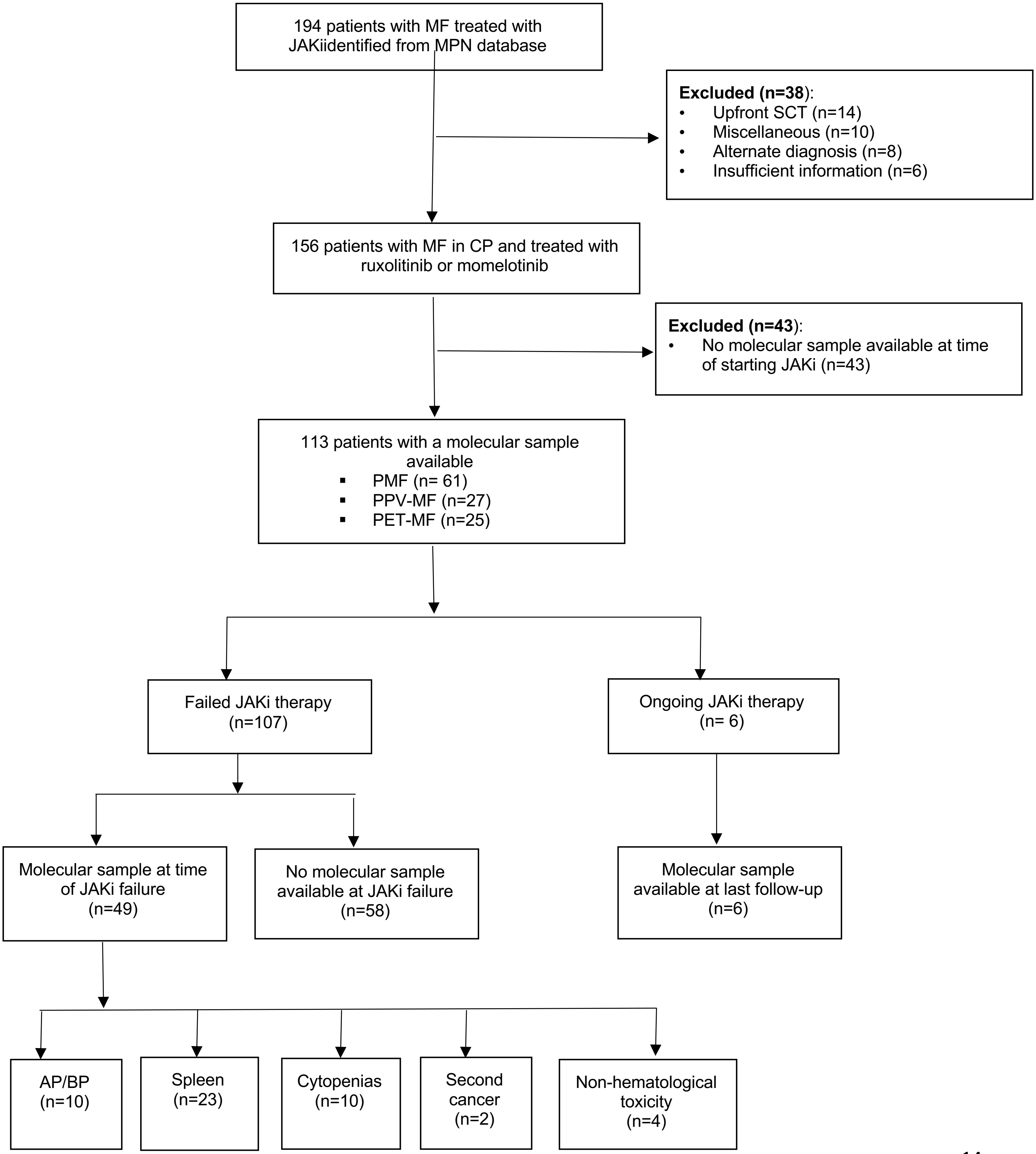

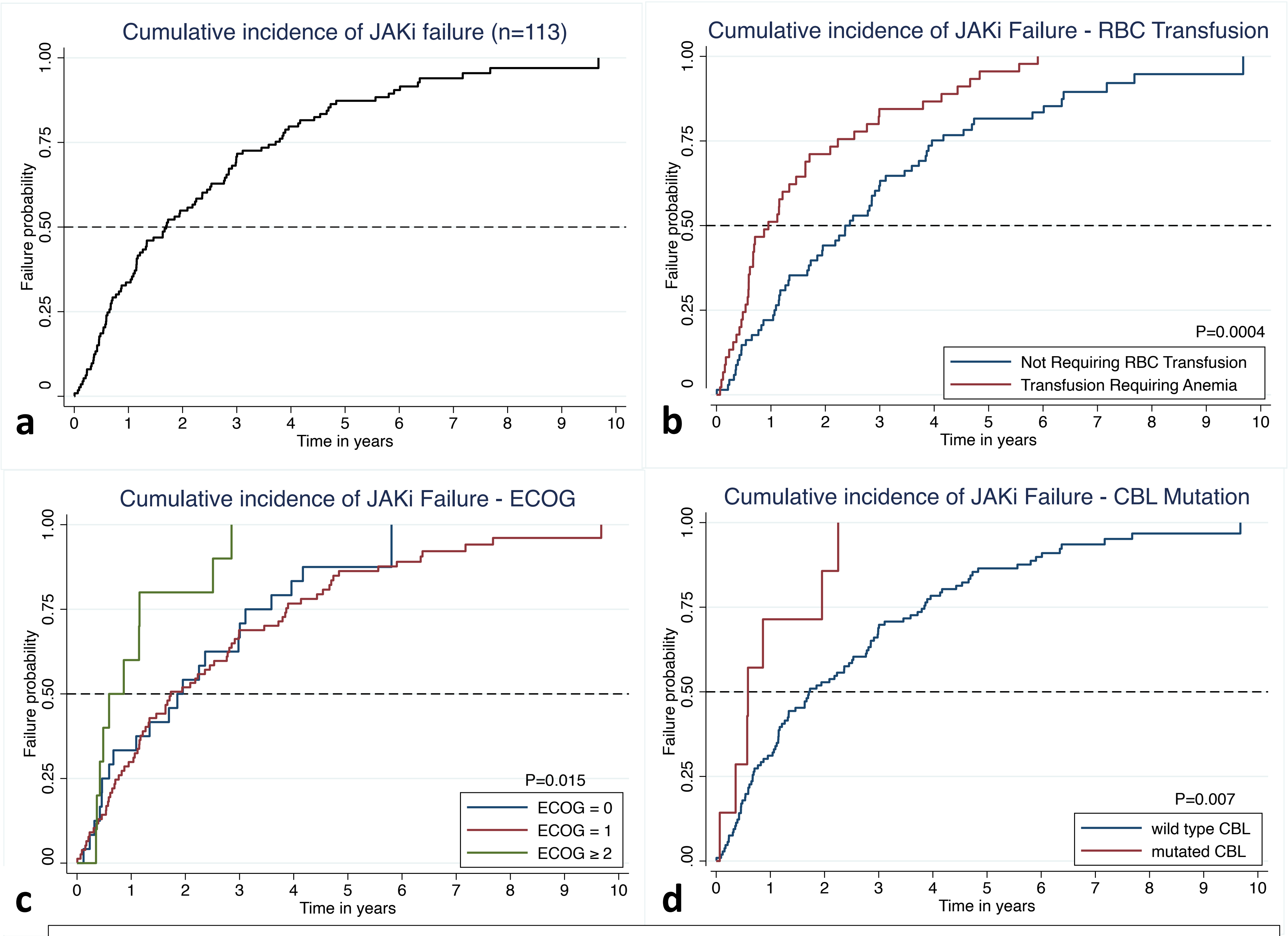


**Supp. Figure S2**: KM curves for cumulative incidence of JAKi failure a) Overall, and by b) transfusion status, c) ECOG performance status, d) CBL mutation status

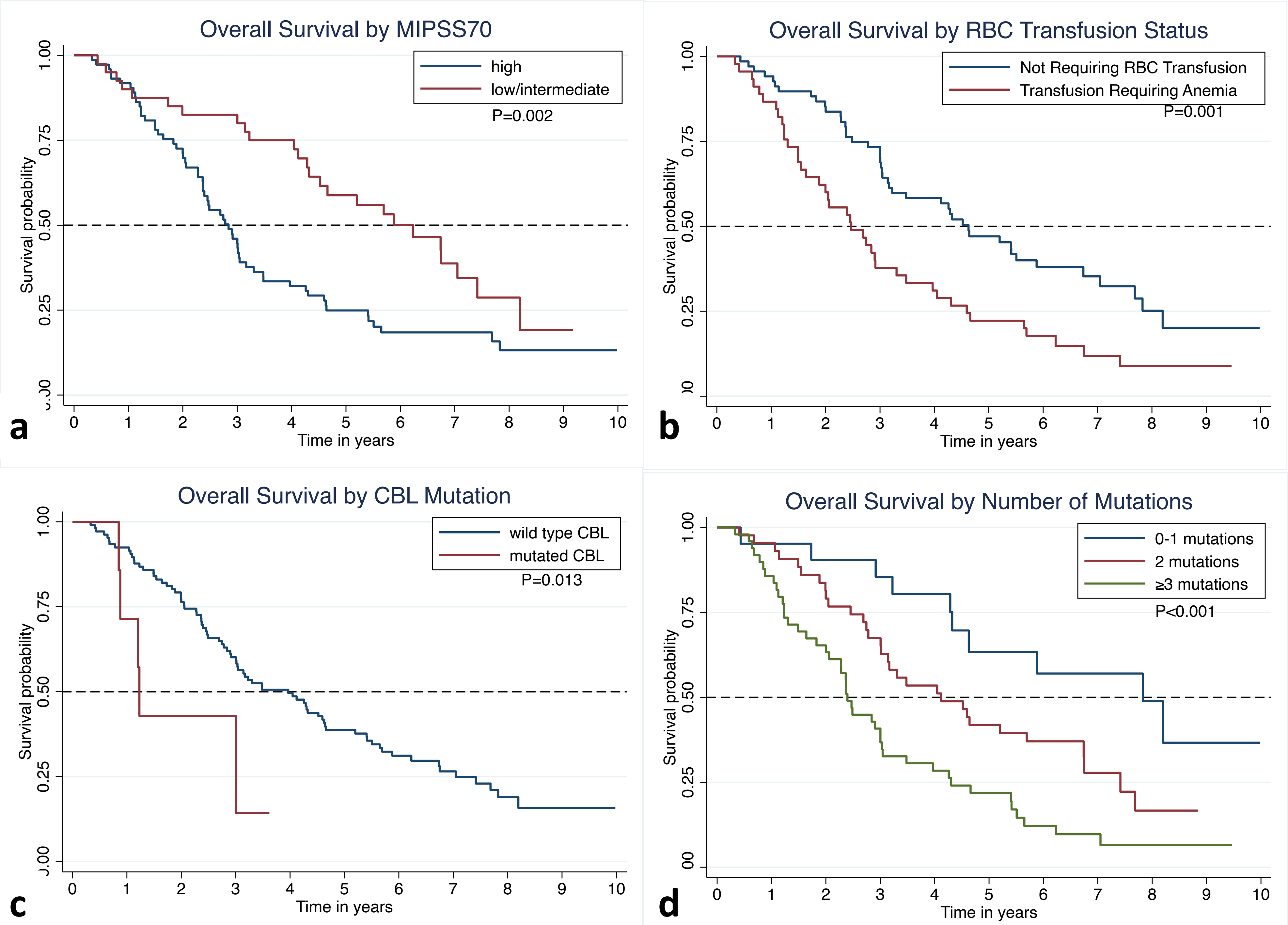


**Supp. Figure S3**: KM curves for overall survival from JAKi start by a) MIPSS70 risk, b) transfusion status, c) CBL mutation status, and d) total number of mutations
